# Nonlinear Relationship Between Lactate/Albumin Ratio and 28‐Day ICU Mortality in Patients With Congestive Heart Failure: Insights Into Inflammatory and Metabolic Interplay

**DOI:** 10.1155/mi/9374199

**Published:** 2026-07-29

**Authors:** Yitong Bian, Ling Wang, Juan Ma, Peng Wu, Ping Jin

**Affiliations:** ^1^ Department of Radiology, First Affiliated Hospital of Xi’an Jiaotong University, Xi’an, Shaanxi, China, xjtu.edu.cn; ^2^ Department of Rehabilitation Medicine, Second Affiliated Hospital, Xi’an Jiaotong University, Xi’an, Shaanxi, China, xjtu.edu.cn; ^3^ Heart Centre and Department of Cardiovascular Diseases, General Hospital of Ningxia Medical University, Yinchuan, Ningxia, China, nxmu.edu.cn; ^4^ Department of Cardiology, Second Affiliated Hospital of Xi’an Jiaotong University, Xi’an, Shaanxi, China, xjtu.edu.cn

**Keywords:** congestive heart failure, eICU‒CRD, ICU mortality, lactate–albumin ratio, nonlinear relationship

## Abstract

**Background:**

Congestive heart failure (CHF) among critically ill patients is a significant cardiovascular disorder that is linked to elevated mortality rates. The lactate/albumin ratio (LAR) is a readily available clinical metric that may provide valuable information regarding the metabolic and nutritional conditions. However, its association with mortality in patients with CHF remains unexplored. This study aimed to investigate the association between LAR and 28‐day intensive care unit (ICU) mortality among critically ill patients with CHF.

**Methods:**

Using the eICU Collaborative Research Database (eICU‐CRD), this study was retrospective and observational. The study included those admitted to the ICU with a preliminary CHF diagnosis. The nonlinear relationship between LAR and 28‐day mortality was assessed using multivariate Cox regression and generalized additive models (GAMs), with confounders adjusted, and the crucial LAR value was identified through threshold effect analysis.

**Results:**

Among 2117 patients diagnosed with CHF, 291 (13.7%) died within 28 days of ICU admission. A nonlinear relationship was observed between the LAR and mortality. For LAR values under 1.65, each unit increment was associated with a 2.41‐fold increase in the likelihood of mortality (*p* < 0.001). Nonetheless, this link was not significant for LARs ≥ 1.65 (HR = 1.14, *p* = 0.111). Subgroup analyses validated the robustness of this nonlinear relationship, except for a significant interaction with the respiratory rate. Mediation analysis revealed that white blood cell (WBC) counts partially mediated the link between LAR and mortality, suggesting potential connections between metabolic disturbances, inflammatory pathways, and adverse outcomes in this population.

**Conclusions:**

LAR was nonlinearly related to 28‐day ICU mortality, identifying a particular turning point in critically ill patients with CHF. These findings suggest that the LAR, a readily available clinical metric, may help identify high‐risk patients with CHF and inform clinical management strategies.

## 1. Introduction

Congestive heart failure (CHF) represents a major cardiovascular disorder marked by impaired organ perfusion and systemic congestion [1]. CHF is a prevalent and growing public health concern driven by an aging population and rising risk factors like hypertension, arrhythmias, and coronary artery disease [2, 3]. The clinical management and prognosis are often complicated and relatively poor, especially for critically ill patients with CHF requiring intensive care unit (ICU) admission [4, 5].

Lactate/albumin ratio (LAR), which is determined by dividing lactate by serum albumin, serves as a readily available clinical metric that may provide insights into the metabolic and nutritional status. Elevated levels of lactate, a product of anaerobic metabolism, have been linked to tissue hypoxia and metabolic dysfunction, which frequently occur in advanced heart failure and are associated with poor outcomes [6–8]. Decreased levels of albumin, a key protein involved in maintaining oncotic pressure and modulating inflammation, have also been related to a poor prognosis in critically ill patients [9, 10]. Albumin functions as a negative acute–phase reactant, with its synthesis suppressed during systemic inflammation while simultaneously serving as an antioxidant and anti‐inflammatory molecule [11, 12]. The LAR has shown promise as a prognostic indicator in several disease conditions, like sepsis, acute pancreatitis, and liver cirrhosis [13–15]. Importantly, recent research has indicated a connection between the LAR and cardiovascular outcomes in patients with myocardial infarction, heart failure, and other heart‐related conditions [16–18]. Emerging evidence suggests that LAR may reflect not only metabolic and nutritional status but also aspects of systemic inflammatory burden, particularly in conditions characterized by tissue hypoperfusion and metabolic stress [19]. In critically ill CHF patients, the interplay between metabolic derangements and inflammatory activation creates a vicious cycle that exacerbates cardiovascular dysfunction and worsens outcomes [20, 21].

Given the potential clinical utility of the LAR, investigating its relationship with outcomes in critically ill patients suffering from CHF is important. The aim of our study was to evaluate the relationship between the LAR and 28‐day all‐cause ICU mortality in patients diagnosed with CHF by utilizing data from the eICU Collaborative Research Database (eICU‐CRD). While the associations between lactate or albumin and mortality have been explored separately in heart failure patients [22, 23], the precise relationships between LAR and outcomes in this high‐risk patient population remain underexplored. By analyzing this association, we hope to provide valuable insights for clinical practice and inform future research directions.

## 2. Methods

### 2.1. Data Source

In this retrospective observational study, the eICU‐CRD, which is an extensive and comprehensive multicenter ICU database, was employed as a vital resource. This database was meticulously developed by the renowned Laboratory for Computational Physiology at MIT in collaboration with Philips Health Care, showcasing a significant advancement in the field of critical care research and data analysis. The database contains clinical data from over 200,000 ICU admissions in 208 United States hospitals during 2014–2015, including demographics, comorbidities, vital signs, lab results, medical use, and outcomes [24].

The eICU‐CRD was deidentified per HIPAA Safe Harbor, and access for research was approved after completing the CITI program and receiving authorization from the PhysioNet committee. The corresponding author was responsible for data extraction (certification number 62661740), and data use followed the Declaration of Helsinki’s ethical standards. This retrospective study was exempt from IRB oversight and received a waiver for informed consent.

### 2.2. Study Population

**Figure 1:**
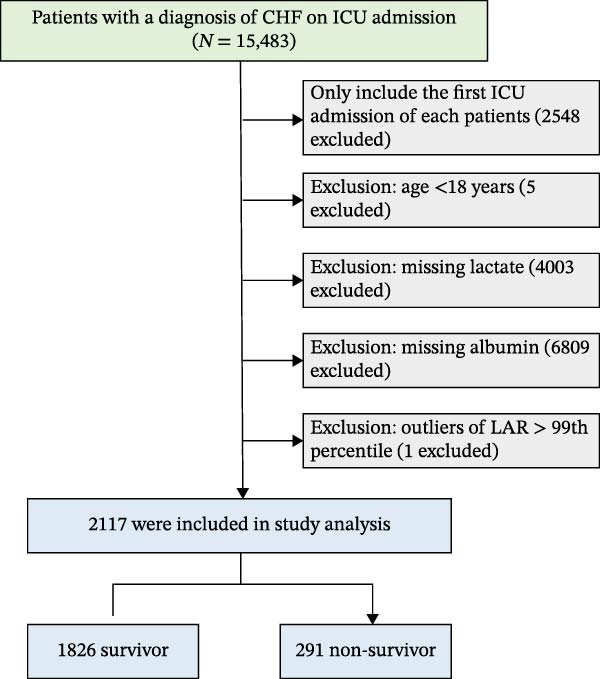
Flow chart of the study population.

The study cohort included ICU patients diagnosed with CHF, which was identified using ICD‐9 code 428 extracted from the “Pre‐existing conditions” field of the diagnosis table in the eICU database. This means that CHF represented an underlying comorbidity rather than necessarily the primary reason for ICU admission. The exclusion criteria were as follows: (1) not first ICU admission, (2) age < 18 years, (3) missing data for lactate, (4) missing data for albumin, and (5) outliers with LAR levels above the 99th percentile. The final study cohort consisted of 2117 patients, including 1826 survivors and 291 (13.7%) nonsurvivors, who were followed for post‐ICU admission to evaluate their all‐cause ICU mortality within 28 days. The flowchart is shown in Figure 1.

### 2.3. Data Extraction

Data were extracted using Structured Query Language (SQL) from the first 24 h post‐ICU admission. Quality control measures were implemented to ensure data reliability, with extreme outliers removed. The following key variables were included: demographics such as gender, age, ethnicity, and body mass index (BMI); comorbidities, including acute myocardial infarction (AMI), arrhythmias, and diabetes mellitus; patient’s vital signs; and severity scores. Laboratory results for glucose, total protein, white blood cells (WBCs), red blood cells (RBCs), platelets (PLTs), lactate, and albumin were obtained from the laboratory tables as the baseline and were documented as the first measurement within 24 h of ICU admission. LAR was determined by dividing the lactate level by the albumin level. The outcome was all‐cause ICU mortality within 28 days after ICU admission.

### 2.4. Outcome Assessment

The primary outcome was 28‐day all‐cause ICU mortality. We selected this endpoint for several reasons: First, 28‐day mortality captures early critical illness deaths most directly influenced by acute metabolic derangements reflected by LAR measured at ICU admission. Second, this timeframe is widely used in critical care research and facilitates comparison with existing ICU prognostic studies. Third, 28‐day ICU mortality minimizes loss to follow‐up compared to longer‐term endpoints and focuses on deaths directly related to the acute illness episode. Finally, early mortality risk stratification is clinically meaningful for intensivists in guiding treatment intensity, resource allocation, and family counseling during the critical care period.

### 2.5. Statistical Analysis

Continuous variables were assessed for normality using the Shapiro–Wilk test. Normally distributed variables are reported as means ± standard deviations (SDs) and compared across LAR tertiles using one‐way ANOVA. Non‐normally distributed variables are reported as medians with interquartile ranges (IQRs) and compared using the Kruskal–Wallis test. Categorical variables are expressed as frequencies and percentages and compared using chi‐square tests or Fisher’s exact test as appropriate. The relationship between LAR and 28‐day ICU mortality was analyzed using univariate and multivariate Cox models, with results as hazard ratios (HRs) and 95% confidence intervals (CIs). The selection of adjusted covariates was based on univariate analysis results, clinical expertise, and existing literature and was further guided by a quantitative criterion requiring a change in the LAR effect estimate of more than 10% or a statistically significant association with the outcome at *p*  < 0.10. Covariates were included as potential confounders in the final models if they met this criterion. Variance inflation factor (VIF) analysis was performed to assess multicollinearity; covariates with VIF > 10 were excluded from the primary model. The following covariates were selected: age, gender, ethnicity, BMI, respiratory rate, heart rate, AMI, arrhythmias, and WBC count. As a sensitivity analysis, the fully adjusted model was further extended to include the APACHE IV score, Acute Physiology Score III, and Glasgow Coma Scale (GCS) score. To enhance statistical power and reduce bias, we created five imputed datasets using multiple imputation with chained equations to address the missing covariates [25, 26]. For sensitivity analysis, repeated analyses were performed on each imputed dataset, and the final results were synthesized by combining the outcomes from each analysis in line with Rubin’s rules [27].

A generalized additive model (GAM) and a piecewise linear regression were used to explore the possible nonlinear connection between the LAR and mortality, with the best model chosen through likelihood ratio testing. Stratified analyses and interaction tests were conducted, with the results expressed as a forest plot. The mediating role of the WBC count in the link between the LAR and mortality was evaluated using mediation analysis. Survival differences between LAR tertiles were compared using Kaplan–Meier curves and log‐rank tests. All statistical analyses were conducted using R software (v4.2.0) (http://www.r-project.org) and the Empower Stats tool (www.empowerstats.com, X&Y solutions, Inc., Boston, MA), with a significance threshold of *p*  < 0.05.

## 3. Results

### 3.1. Baseline Characteristics

Notable differences were found in several demographic and clinical variables grouped by tertiles of LAR, as shown in Table 1. The proportion of males was greater in Tertile 1 (*p* = 0.046), whereas age did not differ across groups. The ethnicity distribution revealed a greater proportion of Caucasians in Tertile 3 (*p*  = 0.047). BMI decreased significantly across tertiles, with Tertile 1 having the highest BMI (*p*  < 0.001). Tertile 2 and Tertile 3 showed a higher proportion of AMI, whereas the incidence of arrhythmias increased with ascending LAR. Disease severity, as indicated by the Acute Physiology Score III, GCS, and APACHE IV scores, was significantly worse in the higher LAR tertiles. Tertile 3 showed increased respiratory and heart rates, whereas the temperature slightly varied across the groups. Tertile 3 had the highest glucose and lactate levels and the lowest albumin and total protein levels. Finally, 28‐day ICU mortality increased with a greater LAR, from 6.56% in Tertile 1 to 23.45% in Tertile 3 (*p* < 0.001).

**Table 1:** Baseline characteristics of the study population according to tertiles of the LAR.

Variables	LAR			*p*‐Value
	Tertile 1 0.09–0.45	Tertile 2 0.45–0.84	Tertile 3 0.84–7.57	
	*n* = 701	*n* = 708	*n* = 708	
Males (%)	340 (48.50%)	316 (44.63%)	297 (41.95%)	0.046
Age (years)	69.30 ± 13.68	69.87 ± 13.67	70.21 ± 13.75	0.456
Ethnicity				0.047
Caucasian (%)	164 (23.40%)	139 (19.63%)	177 (25.00%)	—
Others (%)	537 (76.60%)	569 (80.37%)	531 (75.00%)	—
BMI (kg/m^2^)	29.83 (24.88, 35.85)	28.28 (23.55, 34.00)	27.80 (23.06, 33.34)	<0.001
AMI (%)	53 (7.56%)	86 (12.15%)	82 (11.58%)	0.009
Arrhythmias (%)	225 (32.10%)	255 (36.02%)	315 (44.49%)	<0.001
Diabetes mellitus (%)	162 (23.11%)	177 (25.00%)	171 (24.15%)	0.708
Acute Physiology Score III	49.00 (37.00, 62.00)	53.00 (40.00, 70.00)	66.00 (51.00, 90.00)	<0.001
GCS score	15.00 (11.00, 15.00)	15.00 (11.00, 15.00)	14.00 (9.00, 15.00)	<0.001
APACHE IV score	65.00 (52.00, 78.00)	69.00 (56.00, 85.00)	83.00 (64.00, 105.00)	<0.001
Temperature (°C)	36.42 ± 0.62	36.38 ± 0.69	36.30 ± 0.84	0.012
Respiratory rate (bpm)	28.00 (12.00, 36.00)	31.00 (14.00, 37.00)	32.00 (23.00, 39.00)	<0.001
Heart rate (bpm)	102.00 (83.50, 116.00)	108.00 (91.00, 127.00)	113.00 (96.25, 131.00)	<0.001
MAP (mmHg)	82.53 ± 43.89	79.45 ± 41.83	82.49 ± 48.38	0.339
Glucose (mg/dL)	125.00 (103.00, 159.00)	138.00 (110.00, 178.25)	148.50 (112.20, 202.75)	<0.001
Total protein (g/dL)	6.33 ± 0.81	6.18 ± 0.84	5.90 ± 0.95	<0.001
WBC (cells × 10^9^/L)	9.40 (7.10, 12.80)	12.10 (8.70, 16.52)	13.35 (9.50, 17.70)	<0.001
RBC (M/mcl)	3.60 ± 0.73	3.80 ± 0.74	3.79 ± 0.79	<0.001
PLT (cells × 10^9^/L)	196.32 ± 79.07	201.32 ± 88.69	190.49 ± 92.61	0.076
Lactate (mmol/L)	0.99 (0.70, 1.20)	1.70 (1.40, 2.00)	3.60 (2.70, 5.30)	<0.001
Albumin (g/dL)	3.10 (2.80, 3.40)	2.90 (2.40, 3.20)	2.70 (2.20, 3.10)	<0.001
28‐Day ICU mortality	46 (6.56%)	79 (11.16%)	166 (23.45%)	<0.001

*Note:* Data are presented as mean ± SD for normally distributed continuous variables, median (IQR) for non‐normally distributed continuous variables, or *n* (%) for categorical variables. *p*‐Values were calculated using one‐way ANOVA for normally distributed variables, Kruskal–Wallis test for non‐normally distributed variables, and chi‐square test for categorical variables. The numbers of missing values for the covariates were 98 (4.63%) for BMI, 300 (14.2%) for Acute Physiology Score III, 53 (2.5%) for GCS score, 300 (14.2%) for APACHE IV score, 27 (1.28%) for respiratory rate, 25 (1.18%) for heart rate, 23 (1.09%) for MAP, 76 (3.59%) for glucose, 148 (6.99%) for total protein, 114 (5.38%) for WBC, 102 (4.82%) for RBC, and 219 (10.34%) for PLT.

Abbreviations: AMI, acute myocardial infarction; BMI, body mass index; GCS, Glasgow Coma Scale; LAR, lactate–albumin ratio; MAP, mean arterial pressure; PLT, platelet; RBC, red blood cell; WBC, white blood cell.

The participants were also categorized into survivors and nonsurvivors (Table S1). The nonsurvivor group had a greater proportion of patients in older age and having an AMI and arrhythmias. These patients also had higher Acute Physiology Score III and APACHE IV scores, as well as lower GCS scores. The nonsurvivor group had lower temperature points but higher respiratory rate, heart rate, WBC count, lactate, and LAR values, as well as lower total protein, PLT count, and albumin.

### 3.2. Univariate Analysis for 28‐Day ICU Mortality

Age (HR = 1.02), non‐Caucasian ethnicity (HR = 1.59), a higher APACHE IV score (HR = 1.02), and Acute Physiology Score III (HR = 1.02) were linked to heightened mortality. A lower GCS score (HR = 0.94) and body temperature (HR = 0.75) were inversely associated with mortality. Additionally, when analyzed as continuous variables, lactate levels (HR = 1.22 per mmol/L increase), albumin levels (HR = 0.80 per mmol/L increase), WBC counts (HR = 1.04 per 10^9^/L increase), and LAR (HR = 1.43 per unit increase) were significantly associated with 28‐day ICU mortality, as shown in Table 2.

**Table 2:** Univariate Cox proportional hazard regression for 28‐day ICU mortality in patients with CHF.

Exposure	Characteristics	HR (95% CI)	*p*‐Value
Gender	—	—	0.384
Males	953 (45.02%)	Reference	—
Female	1164 (54.98%)	1.11 (0.88, 1.40)	—
Age (years)	69.80 ± 13.70	1.02 (1.01, 1.03)	<0.001
Ethnicity	—	—	0.003
Caucasian	480 (22.67%)	Reference	—
Others	1637 (77.33%)	1.59 (1.18, 2.15)	—
BMI (kg/m^2^)	28.54 (23.87, 34.31)	0.98 (0.97, 1.00)	0.043
APACHE IV score	71.00 (57.00, 90.00)	1.02 (1.01, 1.02)	<0.001
Acute Physiology Score III	55.00 (42.00, 74.00)	1.02 (1.01, 1.02)	<0.001
GCS score	14.00 (11.00, 15.00)	0.94 (0.91, 0.97)	<0.001
Temperature (°C)	36.37 ± 0.72	0.75 (0.64, 0.88)	0.005
Respiratory rate (bpm)	30.00 (13.00, 37.00)	1.01 (1.00, 1.02)	0.013
Heart rate (bpm)	108.00 (91.00, 126.00)	1.00 (1.00, 1.01)	0.019
MAP (mmHg)	81.49 ± 44.80	1.00 (1.00, 1.00)	0.178
AMI	221 (10.44%)	1.32 (0.95, 1.82)	0.096
Arrhythmias	795 (37.55%)	1.12 (0.89, 1.41)	0.341
Diabetes mellitus	510 (24.09%)	0.98 (0.75, 1.27)	0.858
Glucose (mg/dL)	152.05 ± 65.05	1.00 (1.00, 1.00)	0.991
Total protein (g/dL)	6.13 ± 0.89	0.77 (0.67, 0.88)	0.001
WBC (cells × 10^9^/L)	11.50 (8.10, 15.50)	1.04 (1.02, 1.05)	<0.001
RBC (M/mcl)	3.73 ± 0.76	1.11 (0.95, 1.29)	0.185
PLT (cells × 10^9^/L)	196.02 ± 87.03	1.00 (1.00, 1.00)	0.146
Lactate (mmol/L)	1.70 (1.10, 2.80)	1.22 (1.17, 1.27)	<0.001
Albumin (g/dL)	2.90 (2.50, 3.30)	0.80 (0.66, 0.97)	0.021
LAR	0.61 (0.38, 1.00)	1.43 (1.32, 1.54)	<0.001

*Note:* Characteristics are presented as mean ± SD for normally distributed continuous variables, median (IQR) for non‐normally distributed continuous variables, or *n* (%) for categorical variables.

Abbreviations: CI, confidence interval; HR, hazard ratio.

### 3.3. Relationship Between the LAR and 28‐Day Mortality

In Model 1 (unadjusted), there was a strong link between the LAR and mortality, with an HR of 1.43 (*p* < 0.001) (Table 3). This association remained robust after adjusting for demographic variables in Model 2, with the high LAR tertile showing an HR of 2.94 (*p* < 0.001). Model 3, which was adjusted for extra covariates including BMI, respiratory rate, heart rate, AMI, arrhythmias, and WBC, demonstrated a significant association (HR = 1.43, *p*  < 0.001), with the highest tertile maintaining a strong association with mortality (HR = 2.36, *p*  < 0.001). The trend for an increased risk of mortality with a higher LAR was significant across all of the models (*p* for trend < 0.001).

**Table 3:** Multivariate Cox proportional hazard regression for the association of the LAR with 28‐day ICU mortality.

Exposures	Model 1	Model 2	Model 3
LAR	1.43 (1.32, 1.54) < 0.001	1.43 (1.32, 1.55) < 0.001	1.43 (1.30, 1.57) < 0.001
LAR tertile			
Low	Reference	Reference	Reference
Middle	1.60 (1.11, 2.30) 0.012	1.57 (1.09, 2.26) 0.015	1.35 (0.92, 2.00) 0.128
High	2.95 (2.13, 4.09) < 0.001	2.94 (2.12, 4.07) < 0.001	2.36 (1.65, 3.38) < 0.001
*p* for trend	1.74 (1.49, 2.04) < 0.001	1.74 (1.49, 2.04) < 0.001	1.58 (1.32, 1.88) < 0.001

*Note:* The data are presented as HR (95% CI) *p*‐values. Model 1 had no adjustments. Model 2: adjust for gender, age, and ethnicity. Model 3: adjust for gender, age, ethnicity, BMI, respiratory rate, heart rate, AMI, arrhythmias, and WBC count.

We also employed multiple imputation analysis for missing values of covariates as a sensitivity analysis. Table S2 illustrates the distributions of both the primary and imputed variables. The results of the regression analysis after multiple imputations were in agreement with those of the primary data (Table S3). In the sensitivity analysis further adjusting for APACHE IV score, Acute Physiology Score III, and GCS score, the association between LAR and 28‐day ICU mortality remained statistically significant (LAR per unit: HR = 1.39, 95% CI: 1.22–1.57, *p*  < 0.001; highest vs. lowest tertile: HR = 1.82, 95% CI: 1.24–2.66, *p* = 0.002), confirming the robustness of the primary findings. The results are shown in Table S5.

### 3.4. Nonlinear Relationship Between the LAR and Mortality

**Figure 2:**
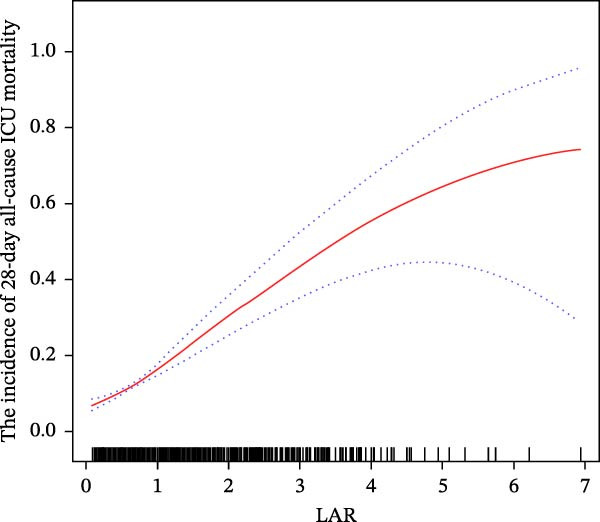
The associations between the LAR and 28‐day ICU mortality in critically ill patients with CHF were fully adjusted with in a generalized additive model (GAM). Estimated values are depicted by the red lines, and their 95% confidence intervals are shown by the blue lines.

Figure 2 illustrates a nonlinear correlation between the LAR and mortality in the fully adjusted model, whereas Table 4 presents a notable threshold effect in this relationship. In the linear model, an increase of one unit in the LAR was correlated with an elevated risk of mortality, as indicated by an HR of 1.42. The nonlinear analysis revealed a turning point at LAR = 1.65, with the risk of mortality significantly increasing when the LAR was below this threshold (HR = 2.41). However, above this threshold (LAR > 1.65), the relationship weakened and became nonsignificant (HR = 1.14). The likelihood ratio test yielded evidence that the nonlinear model delivers a markedly enhanced fit for the data (*p*  < 0.001).

**Table 4:** Threshold effect analysis of the LAR and 28‐day ICU mortality.

Models	Per‐unit increase	
	HR (95% CI)	*p*‐Value
Model I		
One line effect	1.42 (1.30, 1.54)	<0.001
Model II		
Turning point (K)	1.65	
LAR < K	2.41 (1.84, 3.16)	<0.001
LAR ≥ K	1.14 (0.97, 1.33)	0.111
*p*‐Value for LRT test ^∗^	—	<0.001

*Note:* Model I: linear analysis. Model II: nonlinear analysis. Adjusted for gender, age, ethnicity, BMI, respiratory rate, heart rate, AMI, arrhythmias, and WBC. LRT: logarithm likelihood ratio test.  ^∗^Model II is notably distinct from Model I when the *p*‐value < 0.05.

### 3.5. Stratified Analysis and Interaction Test

**Figure 3:**
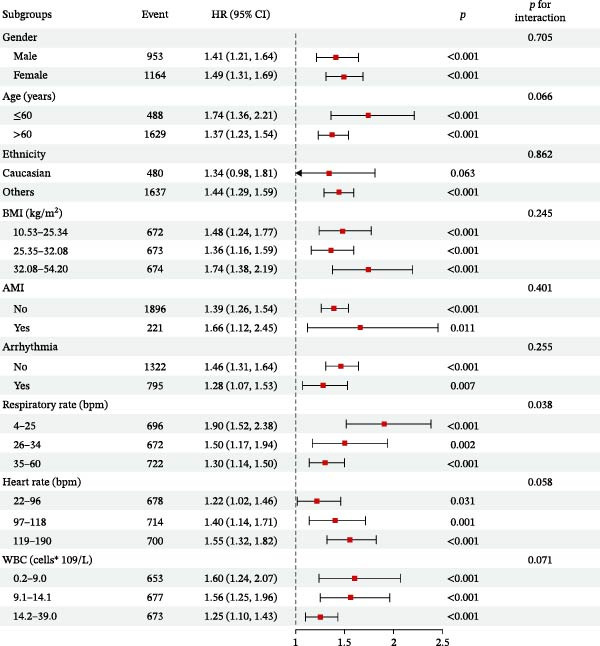
Stratified analysis and interaction test. All stratifications were adjusted for all factors except the stratification component.

**Figure 4:**
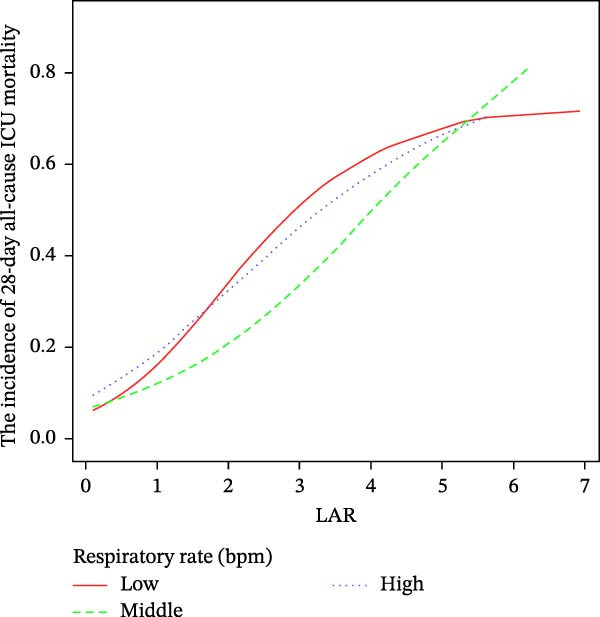
Associations between the LAR and 28‐day ICU mortality according to different respiratory rate groups. LAR: lactate‐to‐albumin ratio. Adjustments were made for gender, age, ethnicity, BMI, AMI, arrhythmias, heart rate, and WBC count.

Stratified analyses were employed based on gender, age, ethnicity, BMI, AMI, arrhythmia, respiratory rate, heart rate, and WBC count (Figure 3). The results consistently revealed that a higher LAR is related to higher 28‐day ICU mortality across most subgroups. However, a significant interaction was identified in the respiratory rate subgroup (*p* for interaction = 0.038). As shown in Figure 4, the HR for the LAR‐to‐mortality association is the highest in the low respiratory rate group (4–25 bpm), moderate in the middle respiratory rate group (26–34 bpm), and lowest in the high respiratory rate group (35–60 bpm).

### 3.6. Correlation Between LAR and WBC Counts

**Figure 5:**
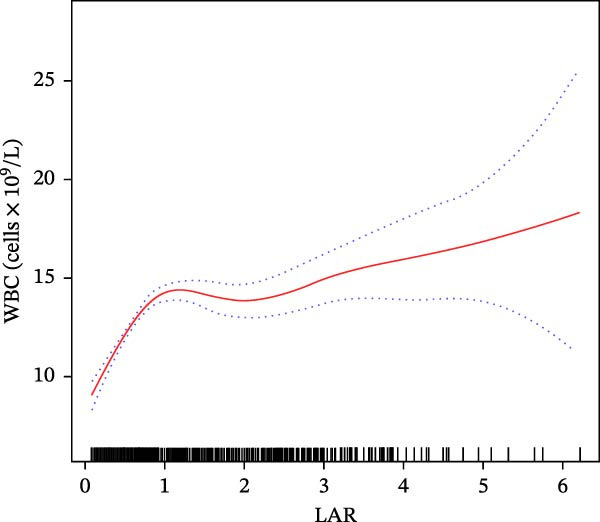
Relationship between LAR and WBC counts in critically ill patients with CHF was fully adjusted using generalized additive model (GAM). Estimated values are depicted by the red lines, and their 95% confidence intervals are shown by the blue lines.

To further explore the relationship between LAR and systemic inflammation, we examined the correlation between LAR and WBC count using smooth curve fitting based on GAM. As shown in Figure 5, a positive correlation was observed between the LAR and WBC count, indicating that higher LAR values are associated with elevated inflammatory markers. This finding supports the notion that LAR reflects not only metabolic disturbances but also aspects of the systemic inflammatory burden in critically ill CHF patients. Subsequently, stratified Cox regression analysis with each WBC tertile using three adjustment models was performed and presented in Table S4. The results consistently showed significant associations between LAR and mortality across all WBC strata and adjustment models (all *p*  < 0.05), further supporting that the LAR‐mortality relationship persists regardless of inflammatory status.

### 3.7. Mediation Analysis

**Figure 6:**
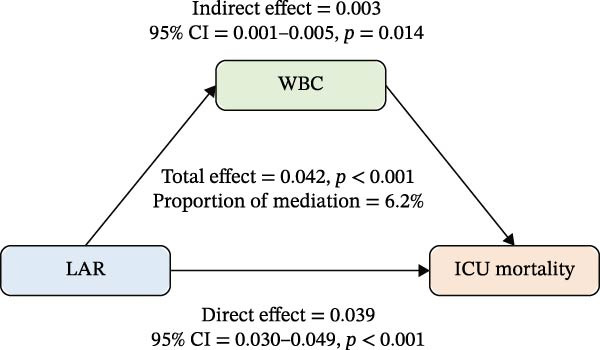
Mediation analysis of the WBC count on the relationship between the LAR and 28‐day ICU mortality after full adjustment.

We further employed mediation analysis to explore whether WBCs function as a mediator in the association between LAR and 28‐day ICU mortality. As shown in Figure 6, WBC counts contributed to 6.2% of the relationship between LAR and mortality after full adjustment.

### 3.8. Survival Curve Analysis

**Figure 7:**
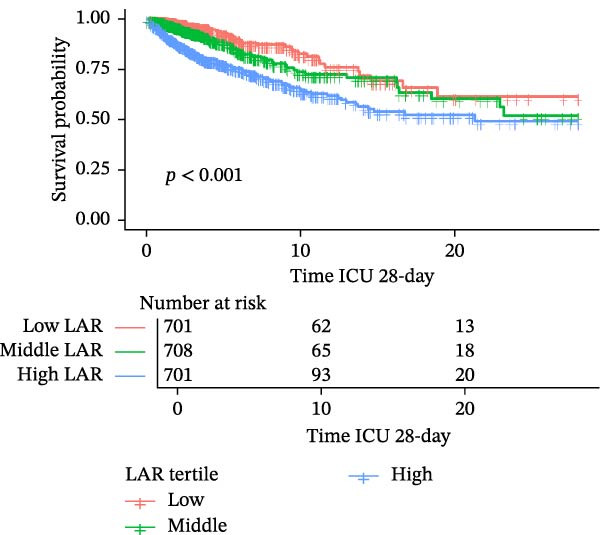
The Kaplan–Meier curves show the survival of critically ill patients with CHF with varying LAR: low (0.09–0.45), middle (0.45–0.84), and high (0.84–7.57).

Differences in the survival probability of critically ill patients with CHF among three groups were determined with Kaplan–Meier survival curves (Figure 7). Patients with higher LAR values (LAR range: 0.84–7.57) experienced notably reduced survival rates at 28 days (*p* < 0.001), indicating the poorest outcomes.

## 4. Discussion

CHF presents an increasing public health challenge, characterized by significant morbidity, mortality, and resource utilization, particularly in the critical care setting [2, 4]. Identifying robust risk stratification tools that can guide management and improve prognostication in this high‐risk patient population is of paramount clinical importance. The present study demonstrated a robust nonlinear relationship between LAR and 28‐day ICU mortality, highlighting a notable threshold effect. Specifically, LAR values falling below 1.65 were correlated with a significant rise in mortality, whereas this strong positive association was attenuated at higher LAR values.

The underlying mechanisms driving this nonlinear LAR–mortality association likely involve the complex interplay between metabolic derangements, nutritional status, and end‐organ perfusion in critical illness [28–30]. Elevated lactate levels, reflected by a greater LAR, may indicate impaired tissue oxygenation and cellular hypoxia, which are hallmarks of advanced heart failure that are strongly linked to adverse outcomes [8, 10, 31]. On the other hand, decreased albumin levels, which also contribute to a greater LAR, can signify inadequate nutritional status and systemic inflammatory activation, both of which are linked to a poor prognosis of patients in heart failure [10, 32, 33]. Hypoalbuminemia in CHF reflects not only malnutrition but also the acute‐phase response, where inflammatory cytokines suppress hepatic albumin synthesis while promoting the production of positive acute–phase proteins [11, 34]. This dual role of albumin as both a nutritional marker and a negative acute–phase reactant underscores the complex interplay between metabolic and inflammatory pathways in critical illness. Conversely, a lower LAR, potentially driven by relatively preserved albumin levels, may denote a more favorable metabolic and nutritional profile, which could confer survival advantages [11]. However, at LAR values ≥ 1.65 (above the identified threshold), the association with 28‐day ICU mortality appeared to plateau (adjusted HR = 1.14, *p* = 0.111), suggesting that at these extremely elevated levels, other severe pathophysiological conditions—such as cardiogenic shock, multiorgan dysfunction, and refractory hemodynamic instability—may become the predominant determinants of mortality [35, 36]. In such circumstances, the metabolic disturbances reflected by LAR likely represent only one component of a multifaceted critical illness state [37]. Similar nonlinear patterns have been observed with other metabolic parameters in critically ill populations, where associations with outcomes may weaken at extreme values due to the overwhelming influence of concurrent severe complications [17, 38].

The findings we obtained align with earlier investigations into the link between LAR and detrimental outcomes in individuals with heart failure [16, 18]. However, our study uniquely highlights the threshold effect of the LAR, with a clear cutoff point beyond which mortality risk increases significantly. This nonlinear association, along with the precise identification of a threshold, adds novel insights into how the LAR might indicate mortality risk meaningfully in critically ill patients suffering from CHF. In addition, our current research focuses on 28‐day ICU all‐cause mortality in critically ill CHF patients during early ICU admission, a vulnerable period characterized by acute hemodynamic instability, severe metabolic derangements, and multiple organ dysfunction, making these patients more complex than the stable outpatient populations examined in previous long‐term outcome studies. Similar studies have shown comparable results but with more emphasis on out‐of‐hospital mortality [18]. Studies have typically examined lactate or albumin alone; however, when these indicators are studied independently, the results may not adequately reflect the interplay of metabolic and nutritional disturbances in patients with CHF [22, 39]. By capturing the complex, multifaceted nature of metabolic and nutritional derangements in critical illness, the LAR metric may offer insights beyond its individual components.

Subgroup analyses further reinforced the robustness of the LAR‐mortality association, with the notable exception of a significant interaction with the respiratory rate. This finding suggests that respiratory status may be an important effect modifier in critically ill patients diagnosed with CHF, underscoring the complex interplay between cardiopulmonary function, metabolic derangements, and clinical outcomes [40].

In individuals exhibiting relatively preserved respiratory function, the LAR may more accurately reflect the degree of tissue hypoxia and lactic acidosis, both of which are notably linked to a negative prognosis in heart failure. When respiratory function is well‐maintained, LAR can act as a sensitive indicator of these metabolic derangements. However, in patients with impaired respiratory function, where the underlying mechanisms of heart failure and respiratory failure are more severe, an increased respiratory rate may dominate the clinical picture, potentially overshadowing the notable association of LAR with mortality. In such cases, the severity of hypoxemia and systemic decompensation may overwhelm the metabolic signals captured by the LAR, leading to a weaker link between the LAR and mortality in the higher respiratory rate subgroup. Zhang et al. [41] reported a J‐shaped link between the respiratory rate and mortality among individuals with CHF and an AMI. These findings highlight the necessity of considering both metabolic and respiratory status when evaluating the mortality of critically ill patients suffering from CHF.

Furthermore, our mediation analysis revealed that the WBC count partially mediated the link between the LAR and 28‐day ICU mortality, suggesting a potential mechanistic link between metabolic disorders, inflammation, and adverse clinical outcomes in this population [19, 21, 42]. This finding aligns with similar results in previous studies demonstrating the significance of inflammation in heart failure [20, 43]. The elevated WBC count associated with higher LAR values may reflect the underlying systemic inflammatory state, which can exacerbate metabolic disturbances and further compromise cardiovascular and end‐organ perfusion, ultimately heightening the mortality risk seen in this cohort of patients.

Our supplementary analyses provide further evidence supporting the link between LAR and systemic inflammation. The observed positive correlation between LAR and WBC counts reinforces the concept that LAR captures aspects of inflammatory burden in addition to metabolic stress. Moreover, the consistent LAR‐mortality associations across different levels of WBC demonstrate that this relationship is robust regardless of the degree of systemic inflammation. These findings collectively suggest that LAR may serve as an integrative metric reflecting the convergence of metabolic derangements and inflammatory activation in critically ill CHF patients.

Survival analysis corroborated the clinical relevance of our findings, demonstrating a substantial rise in the risk of 28‐day ICU mortality linked to low admission LAR. This underscores the potential utility of the LAR as a readily available tool to risk‐stratify critically ill patients with CHF and guide targeted interventions in this high‐risk population.

This study has several limitations. As a retrospective observational analysis, causality cannot be established, and unmeasured confounders (including unadjusted comorbidities such as diabetes severity, specific arrhythmia types, and chronic kidney or liver disease) may have influenced the observed associations. While the outcome and exposure variables were complete for all patients, some covariates had missing values. Although we addressed this using multiple imputations with results consistent across sensitivity analyses, residual bias cannot be entirely excluded if missingness was related to unmeasured factors affecting outcomes. Notably, albumin variability related to fluid status, hepatic function, and inflammatory responses may not be fully captured by the available covariates [11], as some relevant parameters (e.g., C‐reactive protein, available for only 1.77% of patients; detailed fluid balance data) were not consistently documented. Additionally, the data did not include information on the etiology and severity of the CHF diagnoses, which may have provided additional context for interpreting the results. Furthermore, the eICU database encompasses >200 ICUs across diverse US hospitals, which may introduce heterogeneity in clinical practices such as fluid management, CHF treatment protocols, and laboratory measurement timing. Moreover, the primary reason for ICU admission was not captured, and patients may have been admitted for various acute conditions with CHF as a comorbidity; therefore, the observed associations reflect outcomes in a heterogeneous critically ill population with underlying CHF. While this broad representation enhances external validity, center‐level variations were not explicitly modeled and may have influenced the observed associations. The data were collected in 2014–2015, prior to widespread adoption of contemporary CHF therapies such as angiotensin receptor‐neprilysin inhibitors (ARNI), sodium‐glucose cotransporter‐2 (SGLT2) inhibitors, and optimized diuretic strategies. While the pathophysiological mechanisms we examined likely remain relevant, changes in CHF management and absolute outcome rates over the past decade may limit direct comparability to the current practice. Additionally, the WBC count was used in both the covariate and mediator roles. This represents a methodological trade‐off: Adjusting for WBC in the outcome model may simultaneously control for part of the hypothesized inflammatory pathway, potentially attenuating the estimated indirect effect. Future studies employing independently measured inflammatory biomarkers or applying instrumental variable approaches may provide less biased mediation estimates. Finally, the generalizability of the findings to non‐ICU settings or other health care systems outside the United States requires further investigation.

## 5. Conclusions

The present study highlighted a pronounced nonlinear relationship between the LAR and 28‐day ICU mortality in critically ill patients suffering from CHF. LAR values below 1.65 were linked to increased mortality, whereas this strength was attenuated at higher levels. The interaction with respiratory status and the mediating role of the WBC count highlight the complex interplay between cardiopulmonary function, metabolic disorders, inflammation, and outcomes in this population. These findings suggest that the LAR may help to risk‐stratify and guide the management of critically ill patients with CHF, necessitating further research on mechanisms and LAR‐guided interventions.

## Author Contributions

Ping Jin provided the main idea and obtained the certification for data use. Yitong Bian and Ling Wang conducted the data analysis. Yitong Bian wrote the manuscript. Juan Ma and Peng Wu completed the validation. Ping Jin revised the manuscript.

## Funding

This research was supported by the Clinical Research Funds of the First Affiliated Hospital of Xi’an Jiaotong University (Grant XJTU1AF2022LSL‐014), the National Natural Science Foundation of China (Grant 82302854), and the China Postdoctoral Science Foundation (Grant 2025M772070).

## Disclosure

All the authors have reviewed the manuscript.

## Conflicts of Interest

The authors declare no conflicts of interest.

## Supporting Information

Additional supporting information can be found online in the Supporting Information section.

## Supporting information


**Supporting Information** Table S1: The baseline characteristics of participants grouped by survivor and nonsurvivor. Table S2: The distributions of variables with missing data, comparing the observed complete case data set to results from pooling the datasets with imputed variables from multiple imputation. Table S3: The multivariate Cox proportional hazard regression of LAR with 28‐day all‐cause mortality with imputed variables from multiple imputation. Table S4: The multivariate Cox proportional hazard regression for the association of the LAR with 28‐day ICU mortality stratified by WBC tertiles. Table S5: Multivariate Cox proportional hazard regression for the association of LAR with 28‐day ICU mortality (sensitivity analysis with additional adjustment for disease severity scores).

## Data Availability

The raw data are fully available at https://eicu-crd.mit.edu/.
