# Supplementary material for: Nonlinear Relationship Between Lactate/Albumin Ratio and 28‐Day ICU Mortality in Patients With Congestive Heart Failure: Insights Into Inflammatory and Metabolic Interplay
Source: Mediators Inflamm. 2026 Jul 29;2026:9374199. doi: 10.1155/mi/9374199 (PMC13417490; doi:10.1155/mi/9374199)
Supplement: Supplementary file 1 — Supporting Information Table S1: The baseline characteristics of participants grouped by survivor and nonsurvivor. Table S2: The distributions of variables with missing data, comparing the observed complete case data set to results from pooling the datasets with imputed variables from multiple imputation. Table S3: The multivariate Cox proportional hazard regression of LAR with 28‐day all‐cause mortality with imputed variables from multiple imputation. Table S4: The multivariate Cox proportional hazard regression for the association of the LAR with 28‐day ICU mortality stratified by WBC tertiles. Table S5: Multivariate Cox proportional hazard regression for the association of LAR with 28‐day ICU mortality (sensitivity analysis with additional adjustment for disease severity scores). [file MI-2026-9374199-s001.docx]

**Table S1**. Baseline characteristics of participants grouped by survivor and non-survivor.

| Variables | Survivor  n = 1826 | Non-survivor  n = 291 | *P* value |
| --- | --- | --- | --- |
| Males (%) | 995 (54.49%) | 169 (58.08%) | 0.254 |
| Age (years) | 69.48 ± 13.92 | 71.80 ± 12.02 | 0.007 |
| Ethnicity 0.024 | | | |
| Caucasian (%) | 429 (23.49%) | 51 (17.53%) |  |
| Other (%) | 1397 (76.51%) | 240 (82.47%) |  |
| BMI (kg/m^2^) | 28.57 (23.85, 34.25) | 28.22 (23.90, 33.76) | 0.276 |
| AMI (%) | 178 (9.75%) | 43 (14.78%) | 0.009 |
| Arrhythmias (%) | 658 (36.04%) | 137 (47.08%) | <0.001 |
| Diabetes mellitus (%) | 435 (23.82%) | 75 (25.77%) | 0.470 |
| Acute Physiology Score III | 53.00 (40.00, 69.00) | 76.00 (58.75, 104.00) | <0.001 |
| GCS score | 15.00 (11.00, 15.00) | 13.00 (6.00, 15.00) | <0.001 |
| Apache IV score | 68.00 (55.00, 85.00) | 94.00 (74.75, 118.25) | <0.001 |
| Temperature (^o^C) | 36.39 ± 0.69 | 36.20 ± 0.88 | <0.001 |
| Respiratory rate (bpm) | 30.00 (13.00, 37.00) | 33.00 (16.00, 39.00) | 0.012 |
| Heart rate (bpm) | 106.00 (90.00, 125.00) | 115.00 (96.00, 133.00) | <0.001 |
| MAP (mmHg) | 82.13 ± 44.13 | 77.47 ± 48.69 | 0.100 |
| Glucose (mg/dl) | 134.00 (107.00, 179.00) | 143.00 (111.00, 177.00) | 0.473 |
| Total protein (g/dl) | 6.18 ± 0.87 | 5.82 ± 0.91 | <0.001 |
| WBC (cells x 10^9^/L) | 11.04 (8.00, 15.10) | 13.60 (9.60, 18.10) | <0.001 |
| RBC (M/mcl) | 3.72 ± 0.75 | 3.77 ± 0.79 | 0.308 |
| PLT (cells x 10^9^/L) | 197.82 ± 85.26 | 184.73 ± 96.82 | 0.021 |
| Lactate (mmol/L) | 1.60 (1.10, 2.60) | 2.68 (1.50, 5.00) | <0.001 |
| Albumin (g/dl) | 2.90 (2.50, 3.30) | 2.80 (2.30, 3.10) | <0.001 |
| LAR | 0.57 (0.37, 0.91) | 0.97 (0.55, 1.89) | <0.001 |

Data are presented as mean ± SD for normally distributed continuous variables, median (IQR) for non-normally distributed continuous variables, or n (%) for categorical variables. P-values were calculated using one-way ANOVA for normally distributed variables, Kruskal-Wallis test for non-normally distributed variables, and chi-square test for categorical variables. BMI body mass index, AMI acute myocardial infarction, GCS Glasgow Coma Scale, MAP mean arterial pressure, WBC white blood cell, RBC red blood cell, PLT platelets, LAR Lactate-to-albumin ratio.

**Table S2.** Distributions of variables with missing data, comparing the observed complete case data set to results from pooling the datasets with imputed variables from multiple imputation

| Variables | Number (%)  with missing data | Complete case  Median (Min - Max) | Multiple imputation  Median (Min - Max) |
| --- | --- | --- | --- |
| BMI (kg/m^2^) | 98 (4.63%) | 28.54 (10.53-54.20) | 28.80 (10.53-54.20) |
| respiratory rate (bpm) | 27 (1.27%) | 30.00 (4.00-60.00) | 30.00 (4.00-60.00) |
| heart rate (bpm) | 25 (1.18%) | 108.00 (22.00-190.00) | 107.00 (22.00-190.00) |
| WBC (cells x 10^9^/L) | 114(5.38%) | 11.50 (0.20-39.00) | 11.60 (0.20-39.00) |

BMI body mass index, WBC white blood cell.

**Table S3.** Multivariate Cox proportional hazard regression of LAR with mortality with imputed variables from multiple imputation.

| Exposures | HR (95%CI) | P value |
| --- | --- | --- |
| LAR | 1.42 (1.30, 1.54) | <0.001 |
| LAR tertile |  |  |
| Low | Ref |  |
| Middle | 1.45 (0.91, 1.99) | <0.001 |
| High | 2.59 (1.69, 3.49) | <0.001 |

HR hazard ratio, CI confidence interval.

LAR lactate to albumin ratio.

**Table S4.** Multivariate Cox proportional hazards regression for the association of the LAR with 28-day ICU mortality stratified by WBC tertiles.

| Exposures | WBC tertiles | | | Total |
| --- | --- | --- | --- | --- |
|  | Low  (0.2-9.0 cells x 10^9^/L) | Middle  (9.1-14.1 cells x 10^9^/L) | High  (14.2-39.0 cells x 10^9^/L) |  |
| Model 1 | | | | |
| LAR | 1.79 (1.43, 2.24) <0.0001 | 1.55 (1.27, 1.89) <0.0001 | 1.33 (1.18, 1.50) <0.0001 | 1.44 (1.31, 1.57) <0.0001 |
| LAR tertile | | | | |
| Low | Reference | Reference | Reference | Reference |
| Middle | 1.28 (0.65, 2.51) 0.4811 | 1.74 (0.88, 3.44) 0.1107 | 1.20 (0.63, 2.27) 0.5836 | 1.40 (0.96, 2.05) 0.0794 |
| High | 2.29 (1.27, 4.12) 0.0058 | 3.33 (1.80, 6.18) 0.0001 | 2.12 (1.18, 3.82) 0.0119 | 2.57 (1.82, 3.61) <0.0001 |
| *P* for trend | 1.52 (1.13, 2.06) 0.0058 | 1.84 (1.37, 2.47) <0.0001 | 1.56 (1.19, 2.04) 0.0012 | 1.65 (1.40, 1.94) <0.0001 |
| Model 2 | | | | |
| LAR | 1.77 (1.42, 2.22) <0.0001 | 1.66 (1.35, 2.06) <0.0001 | 1.33 (1.18, 1.49) <0.0001 | 1.44 (1.32, 1.57) <0.0001 |
| LAR tertile | | | | |
| Low | Reference | Reference | Reference | Reference |
| Middle | 1.27 (0.64, 2.50) 0.4964 | 1.62 (0.82, 3.21) 0.1630 | 1.16 (0.61, 2.20) 0.6452 | 1.38 (0.95, 2.02) 0.0943 |
| High | 2.32 (1.29, 4.18) 0.0051 | 3.27 (1.76, 6.10) 0.0002 | 2.05 (1.14, 3.70) 0.0168 | 2.55 (1.81, 3.60) <0.0001 |
| *P* for trend | 1.54 (1.14, 2.08) 0.0051 | 1.85 (1.37, 2.50) <0.0001 | 1.54 (1.17, 2.02) 0.0018 | 1.65 (1.39, 1.94) <0.0001 |
| Model 3 | | | | |
| LAR | 1.69 (1.32, 2.17) <0.0001 | 1.65 (1.32, 2.06) <0.0001 | 1.28 (1.12, 1.45) 0.0002 | 1.44 (1.31, 1.58) <0.0001 |
| LAR tertile | | | | |
| Low | Reference | Reference | Reference | Reference |
| Middle | 1.22 (0.60, 2.49) 0.5791 | 1.51 (0.74, 3.09) 0.2527 | 1.09 (0.56, 2.12) 0.8080 | 1.37 (0.92, 2.03) 0.1204 |
| High | 2.09 (1.10, 3.95) 0.0243 | 2.87 (1.49, 5.53) 0.0016 | 1.87 (1.00, 3.48) 0.0484 | 2.41 (1.68, 3.46) <0.0001 |
| *P* for trend | 1.46 (1.05, 2.01) 0.0232 | 1.74 (1.27, 2.38) 0.0006 | 1.48 (1.11, 1.97) 0.0075 | 1.59 (1.34, 1.90) <0.0001 |

The data are presented as HR (95% CI) *P-*values.

Model 1 had no adjustments.

Model 2 adjust for gender, age, and ethnicity.

Model 3: adjust for gender, age, ethnicity, BMI, respiratory rate, heart rate, AMI and arrhythmias.

**Table S5.** Multivariate Cox proportional hazards regression for the association of LAR with 28-day ICU mortality (sensitivity analysis with additional adjustment for disease severity scores)

| Exposures | Model 1 | Model 2 | Model 3 |
| --- | --- | --- | --- |
| LAR | 1.43 (1.32, 1.54) <0.001 | 1.43 (1.32, 1.55) <0.001 | 1.39 (1.22, 1.57) <0.0001 |
| LAR tertile |  |  |  |
| Low | Ref | Ref | Ref |
| Middle | 1.60 (1.11, 2.30) 0.012 | 1.57 (1.09, 2.26) 0.015 | 1.19 (0.79, 1.80) 0.4078 |
| High | 2.95 (2.13, 4.09) <0.001 | 2.94 (2.12, 4.07) <0.001 | 1.82 (1.24, 2.66) 0.0023 |
| *P* for trend | 1.74 (1.49, 2.04) <0.001 | 1.74 (1.49, 2.04) <0.001 | 1.38 (1.14, 1.66) 0.0008 |

The data are presented as HR (95%CI) *P-*values.

Model 1had no adjustments.

Model 2 adjust for gender, age, and ethnicity.

Model 3 (Additional): additionally adjusted for BMI, respiratory rate, heart rate, AMI, arrhythmias, WBC count, APACHE IV score, Acute Physiology Score III, and GCS score.
